# The significance of lumican expression in ovarian cancer drug-resistant cell lines

**DOI:** 10.18632/oncotarget.20169

**Published:** 2017-08-10

**Authors:** Andrzej Klejewski, Karolina Sterzyńska, Karolina Wojtowicz, Monika Świerczewska, Małgorzata Partyka, Maciej Brązert, Michał Nowicki, Maciej Zabel, Radosław Januchowski

**Affiliations:** ^1^ Department of Nursing, Poznań University of Medical Sciences, Poznań, Poland; ^2^ Department of Obstetrics and Womens Diseases, Poznań University of Medical Sciences, Poznań, Poland; ^3^ Department of Histology and Embryology, Poznań University of Medical Sciences, Poznań, Poland; ^4^ Division of Infertility and Reproductive Endocrinology, Department of Gynecology, Obstetrics and Gynecological Oncology, Poznań University of Medical Sciences, Poznań, Poland; ^5^ Department of Histology and Embryology, Wrocław Medical University, Wrocław, Poland

**Keywords:** drug resistance, ovarian cancer, lumican, collagen

## Abstract

**Purpose:**

The aim of the present study is to determine the expression of LUM in drug-resistant ovarian cancer cell lines.

**Methods:**

Doxorubicin- (DOX), topotecan- (TOP) and vincristine- (VIN) resistant variants of the W1 ovarian cancer cell line were used in this study. We used quantitative real-time polymerase chain reactions to determine LUM mRNA levels. Protein expression was detected using Western blot and immunocytochemistry assays. Protein glycosylation was investigated using PGNase F digestion. Immunohistochemistry assays were used to determine protein expression in ovarian cancer patients.

**Results:**

We observed increased expression of LUM in drug-resistant cell lines at both the mRNA and the protein level. The most abundant LUM expression was observed in TOP-resistant cell line. We observed LUM bands that corresponded to different molecular masses, and the most abundant LUM form was the secreted form, which had a mass of 50 kDa. Double immunofluorescence analysis showed co-expression of LUM and COL3A1 as well as the presence of extracellular COL3A1 in the TOP-resistant cell line. Finally, we detected the LUM protein in ovarian cancer tissue.

**Conclusion:**

The expression of LUM in cytostatic-resistant cell lines suggests its role in drug resistance. The co-expression of LUM and COL3A1 indicates the significance of LUM in collagen fibre assembly. Expression in ovarian cancer tissue suggests that LUM can play a role in ovarian cancer pathogenesis in ways similar to other cancers.

## INTRODUCTION

Resistance to cytotoxic drugs, either inherent resistance or, more often, resistance acquired during treatment, is one of the most important reasons for low chemotherapy effectiveness in cancer patients [1]. The mechanisms of cancer drug resistance can be divided into cellular and cancer tissue-specific groups. Cellular mechanisms of drug resistance are well described. The most important are decreased accumulation of the drug in the cancer cell, faster inactivation of the drug, and demand of faster repair of DNA and other cellular components by the drugs. The most important mechanism of drug resistance at the cellular level, which is more often described in the literature, is the expression of drug transporters from the ABC family, such as glycoprotein P (P-gp) and BCRP (breast cancer resistant protein) [2-4]. In contrast, much less is known about cancer tissue-specific mechanisms of resistance to cytotoxic agents. Diffusion of drug molecules in tumour tissue is limited by a dense cellular structure [5] and growth-induced solid stress [6]. Expression of extracellular matrix (ECM) components, such as proteoglycans and collagens, in cancer cells and stroma can also limit the diffusion rates of anti-cancer agents [7]. ECM components interact with cancer cells and induce their resistance by inhibiting sensitivity to apoptosis [8]. This kind of resistance to drugs is designated as cell adhesion-mediated drug resistance (CAM-DR) [9, 10] and was noted both *in vitro* [11] and *in vivo* [12]. Expression of ECM components was observed not only in tumour tissue but also in drug-resistant ovarian cells [13-15] and breast cancer cell lines [16].

Lumican (LUM) is a member of the small leucine-rich proteoglycan (SLRP) family [17]. Amino acid sequence data indicate that the central region of the protein contains four asparagine residues that are N-linked with keratan sulphate (KS) or oligosaccharides [18, 19]. The molecular mass of the core protein is 38 kDa and can increase to 55-57 kDa in the glycoprotein form and 50-100 kDa, or even higher, in the proteoglycan form [20]. Different forms of LUM are differentially expressed in tissue. The non-glycosylated form of LUM was observed in lung fibroblasts [21], the glycoprotein form was detected in the dermis [20] and the KS form of LUM was found in corneal stroma [22]. LUM co-localizes with fibrillar collagen and plays an important role in the assembly and diameter of collagen fibres [23].

The expression of LUM was reported in many cancers. In breast tumours, the expression of LUM was detected at the mRNA and protein levels, and it was concluded that LUM is the most important proteoglycan in breast tumours [24]. LUM expression in advanced colorectal cancer with nodal metastasis was detected in 62.7% of patients and was correlated with the spread of lymph node metastasis, the depth of tumour invasion and significantly lower survival rates of patients [25]. In lung adenocarcinoma (ADC) and squamous cell carcinoma (SCC), the expression pattern and glycosylated form of LUM in cancer cells and in stromal tissue correlated with the aggressiveness of ADC and SCC [21]. The expression of LUM in stromal tissues correlated with shorter survival times of pancreatic cancer patients [26]. LUM was also identified as a cisplatin- (CIS) resistant related gene in head and neck squamous cell carcinoma (HNSCC). Furthermore, downregulation of the LUM gene in the HNSCC cell line resulted in an increased sensitivity to CIS [27]. To gain a better understanding of the role of LUM in drug resistance development, we used an ovarian cancer model, which is the most lethal gynaecological malignancy [28]. Although ovarian cancer is one of the most treatable solid tumours, at the beginning of treatment, it can develop drug resistance in most cases. The first-line chemotherapy regimen for ovarian cancer treatment consists of CIS and paclitaxel (PAC) [28]. In the second line of chemotherapy treatment, doxorubicin (DOX), topotecan (TOP) and gemcitabine are mainly used in cases of platinum-resistant disease [29, 30].

In this study, we used DOX-, TOP- and vincristine- (VIN) resistant ovarian cancer cell lines. DOX and TOP inhibit DNA topoisomerase I and II, respectively, which are enzymes that regulate the overwinding or underwinding of the DNA helix [31]. Blocking DNA topoisomerase activities by DOX or TOP results in the formation of irreversible covalent cross-links between the topoisomerase and DNA, leading to DNA breakage and, eventually, cell death [31]. Another anti-cancer drug that is not used in ovarian cancer therapy is VIN. VIN binds to tubulin and halts the separation of chromosomes during mitosis, which results in cell apoptosis [32]. The main cellular mechanism of resistance to DOX and VIN seems to be associated with the expression of P-gp [2, 4, 33]. Resistance to TOP is mainly based on BCRP expression [2, 3].

Our previous microarray results indicated that LUM was overexpressed in three of six drug-resistant ovarian cancer cell lines [14]. In this study, we compared the expression of LUM at mRNA and protein levels in DOX- (W1DR), VIN- (W1VR) and TOP-resistant (W1TR) cell lines and in their corresponding media. We also showed that LUM can be involved in the response to TOP at the beginning of treatment. Eventually, analysis of paraffin sections confirmed the presence of LUM in ovarian cancer tissue.

## RESULTS

### Analyses of LUM gene expression in drug-resistant ovarian cancer cell lines

**Figure 1:**
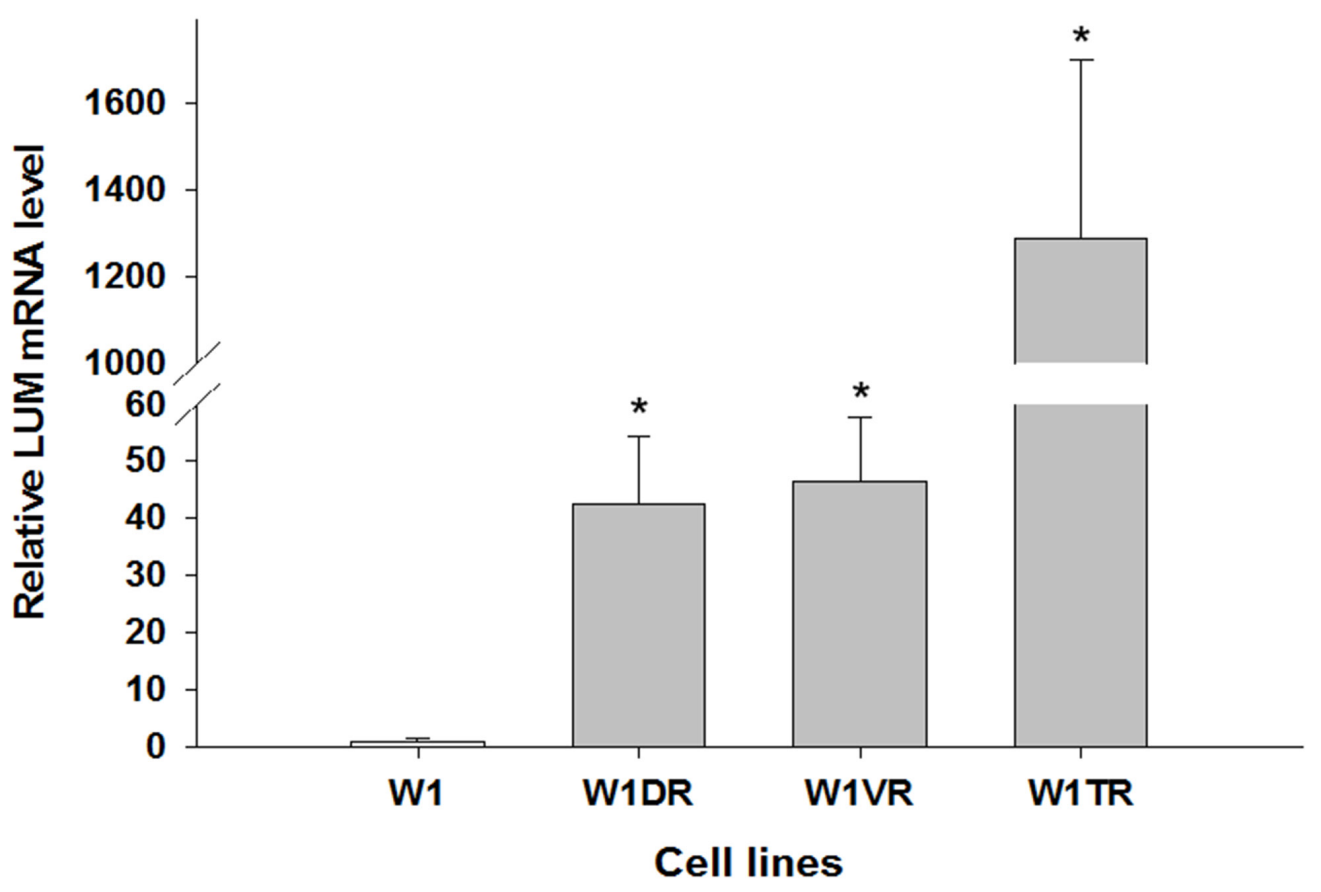
Expression analysis (Q-PCR) of the *LUM* gene This figure presents relative gene expression in resistant cell lines (W1DR, W1TR and W1VR grey bars) with respect to the W1 cell line (white bars), which is assigned a value of 1. Values were considered significant at *p < 0.001.

To determine whether the development of drug resistance in W1 drug-resistant sublines is associated with *LUM* overexpression, expression of the *LUM* mRNA was assessed. We observed a statistically significant increase of the *LUM* transcript in DOX- (W1DR), VIN- (W1VR) and TOP-resistant (W1TR) cell lines (*p* < 0.001) (Figure 1). However, the expression of *LUM* was variable in these cell lines. We observed approximately 40-fold higher transcript levels in the W1DR and W1VR cells compared to the control. Expression in the W1TR cells increased over 1200-fold in comparison to the W1 cell line.

### Immunofluorescence of the LUM protein expressed in resistant cell lines

**Figure 2:**
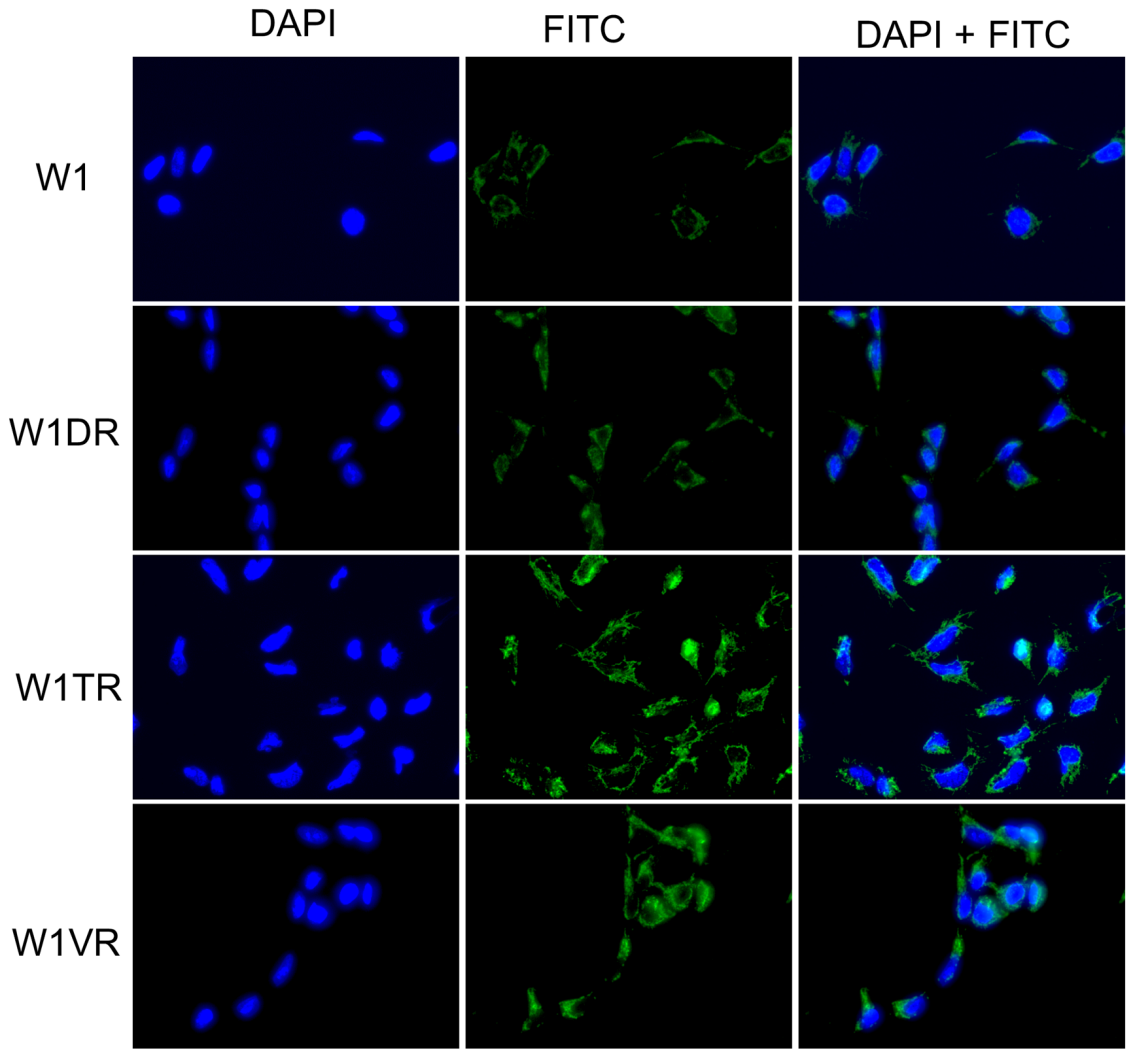
Immunofluorescence visualization of LUM expression in the W1, W1DR, W1TR and W1VR cell lines LUM was detected using the anti-LUM antibody and an MFP488-conjugated secondary antibody (green). To visualize the cell nuclei, the cells were mounted with a DAPI-containing mounting medium (blue).

To confirm the presence of the LUM protein in the investigated cell lines, we performed fluorescence analysis of its expression in W1 and drug-resistant cell lines. A low fluorescence signal was present in the W1 cell line. In the W1DR and W1VR cell lines, we observed some increase in fluorescence intensity. A clear increase in fluorescence signal was observed in the W1TR, TOP-resistant cell line (Figure 2).

### Western blot analysis of LUM

**Figure 3:**
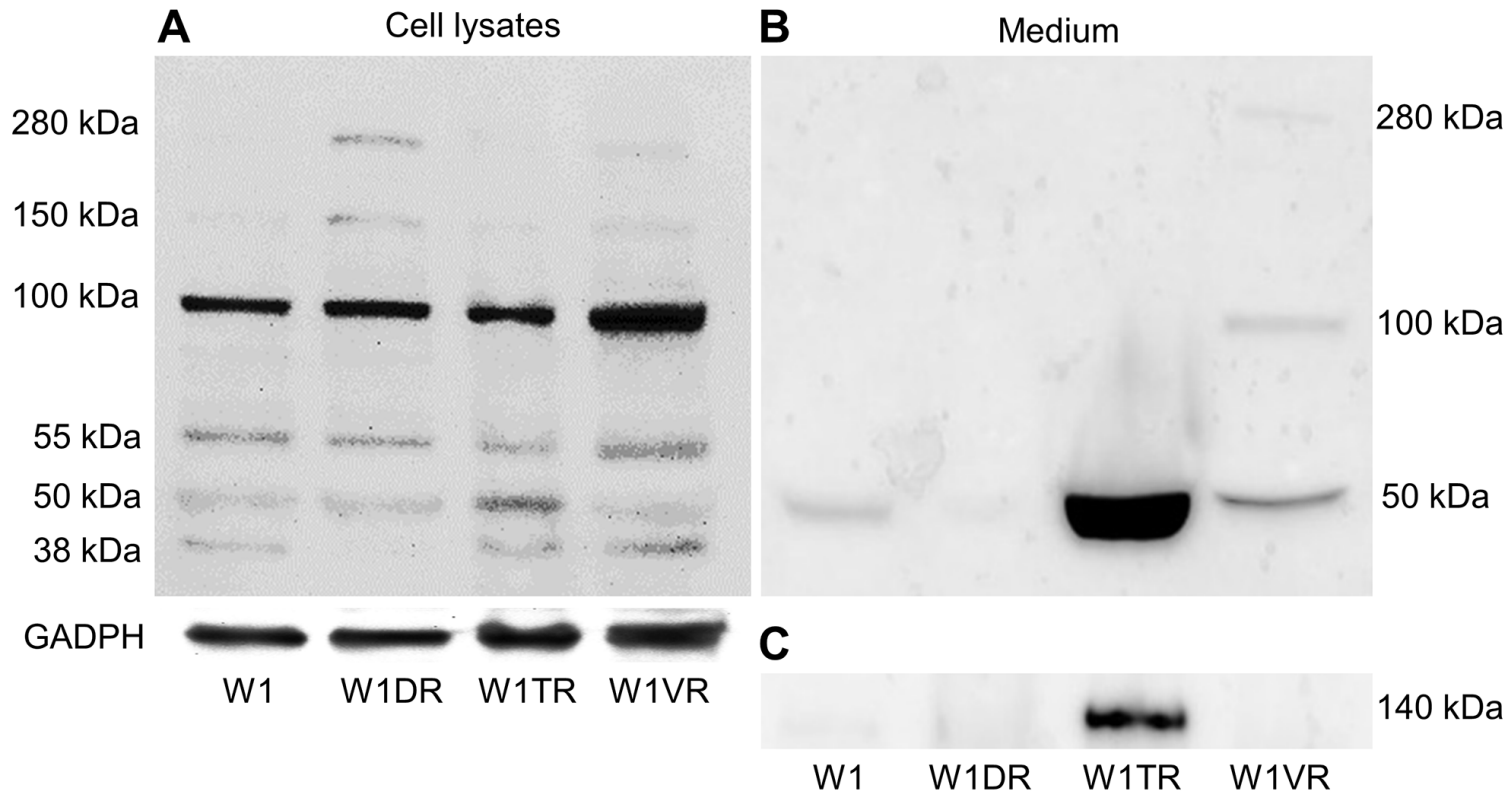
LUM protein expression analysis in the W1 and drug-resistant cell lines W1DR, W1TR and W1VR (A) and their corresponding media (B) COL3A1 expression analysis in cell culture media **(C)**. The cellular proteins and proteins isolated from the media were separated using 7% PAGE and transferred to a PVDF membrane, which was then immunoblotted with either primary Ab or HRP-conjugated secondary Ab. A primary anti-GADPH Ab was used as a loading control for the cell lysates.

To be certain that expression of LUM was also increased at the protein level, we decided to confirm its expression using Western blot analysis (Figure 3A, 3B). Several bands were detected in the sensitive and resistant cell lines. Expression of the core protein with a molecular mass of 38 kDa was present in lysates from the W1, W1TR and W1VR cell lines. In the W1TR cell line, we observed increased expression of a band with a molecular mass of 50 kDa. LUM that had a molecular mass of 55 kDa was present in all resistant cell lines. However, the most abundant form of LUM that was observed had a molecular mass of 100 kDa. This form was upregulated in the W1VR cell line. We also observed very low expression of the forms with masses of 150 kDa and approximately 280 kDa in the W1DR and W1VR cell lines. In media, we observed strong overexpression of a band corresponding to 50 kDa in the W1TR cell line. This band was also slightly overexpressed in the W1VR cell line but was not present in the W1DR cell line. We also observed the presence of bands corresponding to approximately 100 kDa and 280 kDa in the W1VR cell line. Most of the LUM expressed was present in the media. Detection of LUM in the media was possible after a brief time exposure. In contrast, detection in cell lysates required much longer exposure. In addition, in the W1TR cell line, we observed the presence of secreted COL3A1 (Figure 3C).

### Enzymatic deglycosylation of LUM

**Figure 4:**
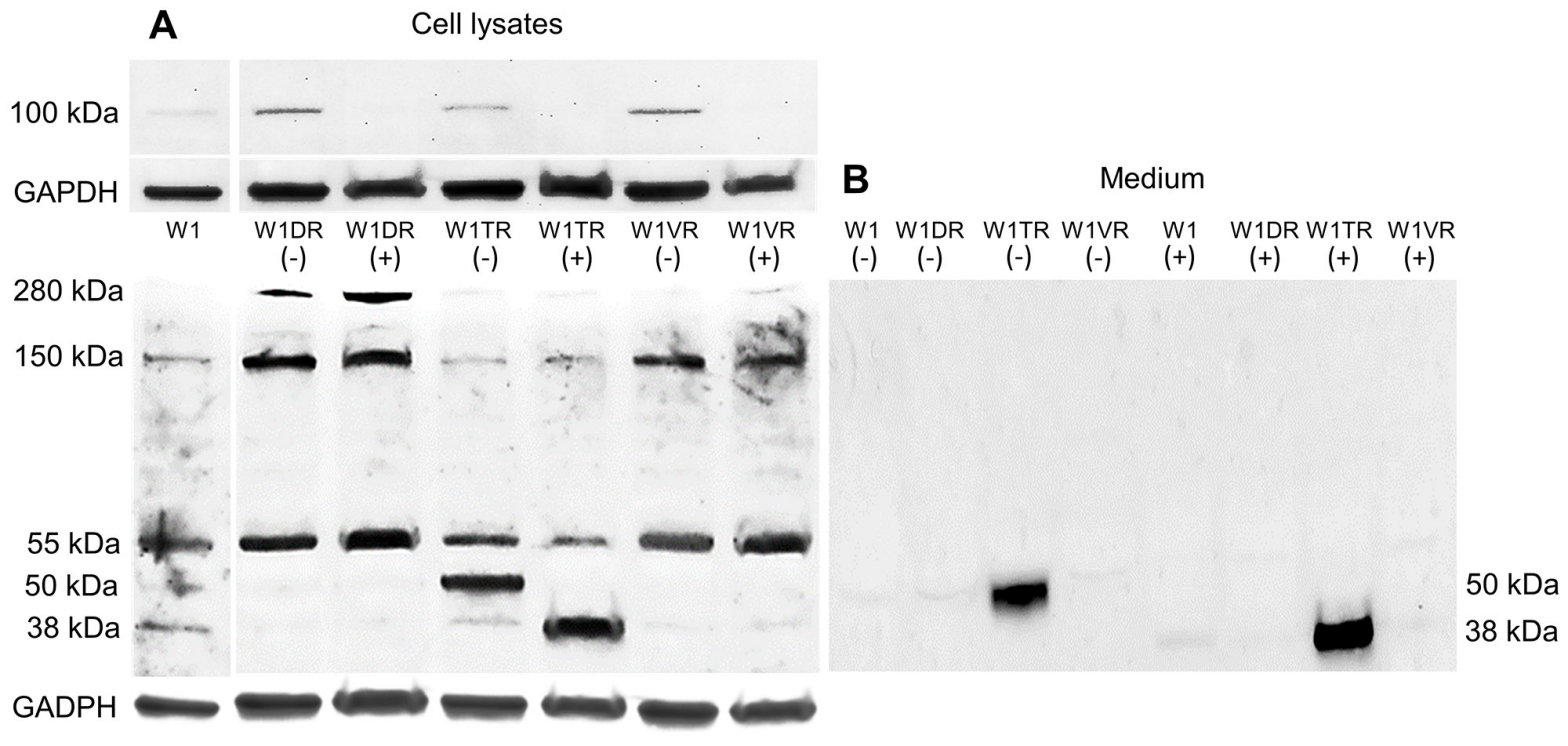
Deglycosylation of LUM with PGNase F in cell lysates (A) and proteins isolated from media (B) The cellular proteins and proteins isolated from media were treated (+) or not treated (-) with PNGase F and separated using 7% PAGE and transferred to a PVDF membrane, which was then immunoblotted with either primary Ab or HRP-conjugated secondary Ab. A primary anti-GADPH Ab was used as a loading control for the cell lysates. Separate exposures were used to identify `bands corresponding to masses of approximately 100 kDa because of low band intensity.

According to the literature data, the molecular mass of the core LUM protein is 38 kDa [17]. Because the N-glycosylated form of LUM was reported previously in pancreatic cancer, we checked whether PGNase F digestion could decrease the molecular mass of LUM. We did not observe any changes in the number of bands that had molecular masses of 280 kDa or 150 kDa after PGNase F digestion. In contrast, bands with 100 kDa were dispersed in all resistant cell lines after digestion. No changes in bands with a mass of 55 kDa after PGNase F treatment were observed. However, in the W1TR cell line, we observed the disappearance of a band with a mass of 50 kDa and the appearance of a band with a mass of 38 kDa, corresponding to core the protein (Figure 4A). An identical result was observed for protein isolated from the W1TR cell line medium (Figure 4B).

### Double immunofluorescence

**Figure 5:**
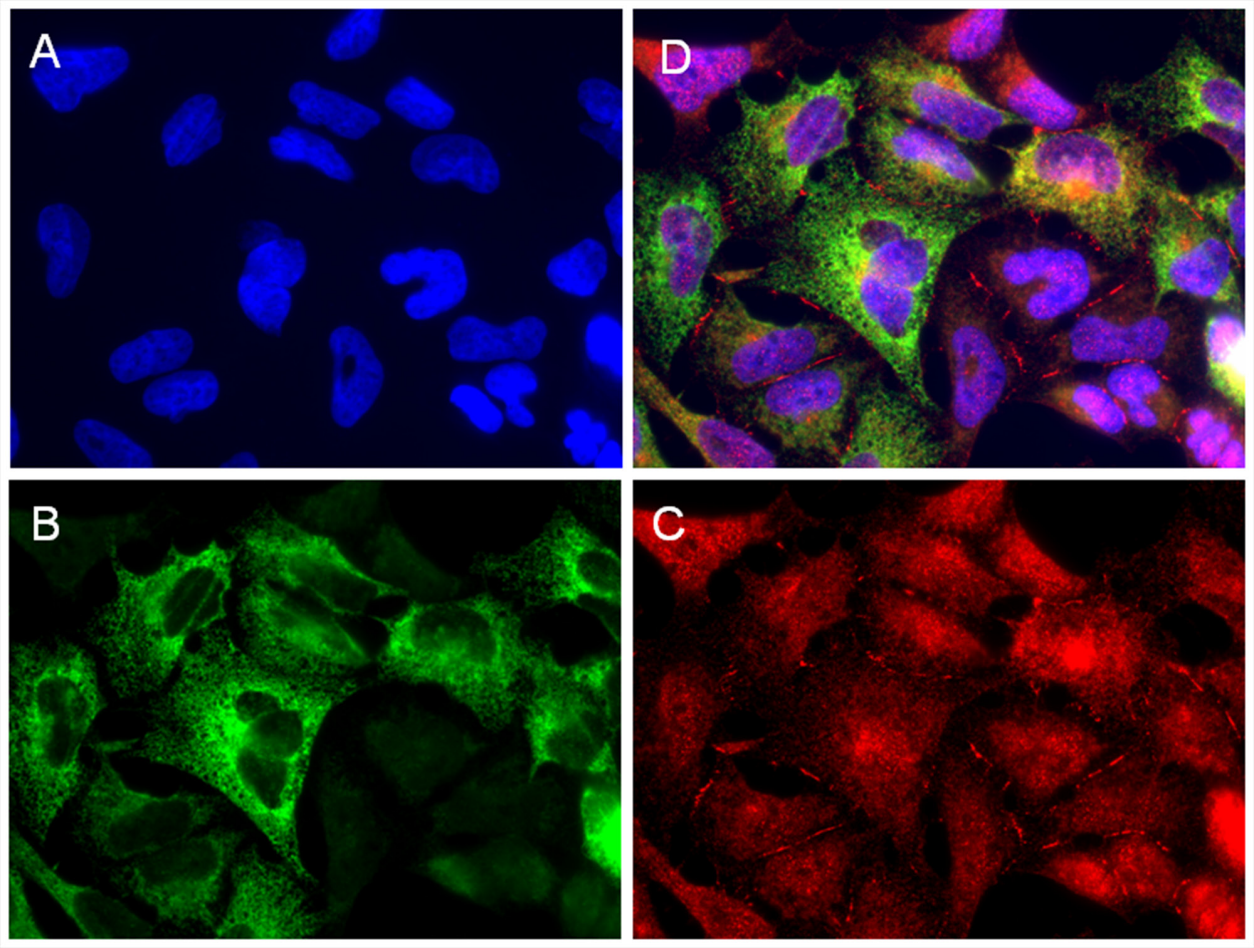
Immunofluorescence visualization of LUM and COL3A1 co-expression in the W1TR cell line. LUM was detected using the anti-LUM antibody and a Cy3-conjugated secondary antibody (red) **(C)**. COL3A1 was detected using the anti-COL3A1 antibody and an MFP488-conjugated secondary antibody (green) **(B)**. To visualize the cell nuclei, the cells were mounted with a DAPI-containing mounting medium (blue) **(A)**. All channels merged **(D)**. Objective 63x.

The double immunofluorescence assay showed co-expression of LUM (red) and COL3A1 (green) in the W1TR cell line. We observed uniform expression of LUM in the whole cell population with more intensive expression around the nuclei and in the cell membranes. The expression of COL3A1 in the same type of cell line was more diverse. We could observe intensive fluorescence in the cytoplasm of some cells whereas others were negative for the same antigen (Figure 5).

### Analysis of LUM gene expression in response to TOP treatment

**Figure 6:**
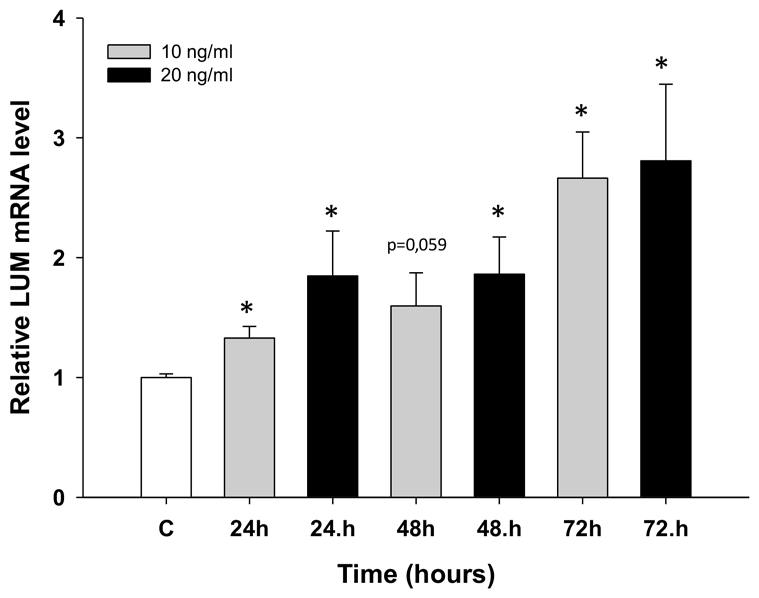
Expression analysis (Q-PCR) of the *LUM* gene in the W1 cell line after TOP treatment This figure presents relative gene expression in treated cells (TOP – 10 ng/ml: grey bars, TOP – 20 ng/ml: black bars) with respect to untreated cells (white bars) at different time points. Untreated cells were assigned a value of 1. Values were considered significant at *p < 0.05.

Next, we wanted to check whether *LUM* expression could be involved during early responses to TOP treatment. The W1 cell line was treated with low concentrations of TOP (10 ng/ml and 20 ng/ml) for 24, 48 and 72 hours. We observed concentration- and time-dependent (*p* < 0.05 except for 10 ng/ml at 48 h) increases in LUM transcript levels after TOP treatment (Figure 6).

### Immunohistochemistry of LUM in ovarian cancer tissue

**Figure 7:**
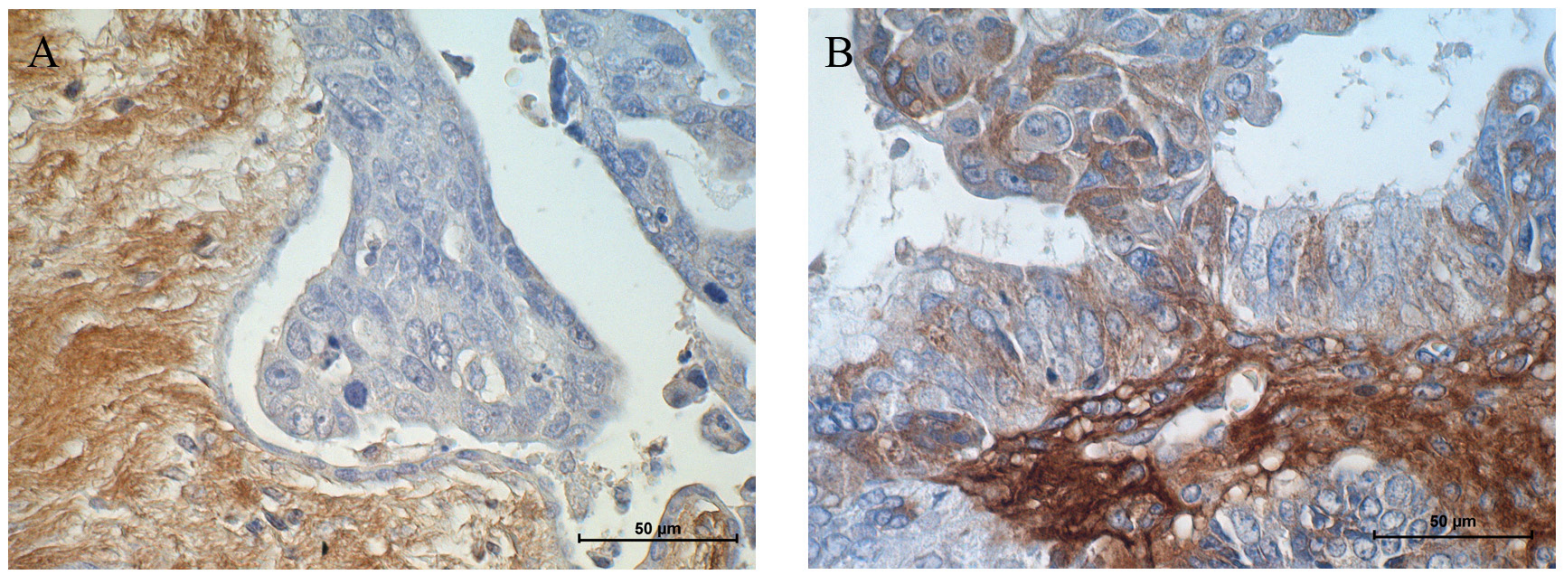
Immunohistochemical expression of LUM in **(A)** stroma of a serous adenocarcinoma patient and **(B)** stroma and cancer cells of an endometrioid adenocarcinoma patient. Sections were counterstained with haematoxylin. Scale bar = 50 μm.

Immunohistochemical analysis of the LUM protein was performed in ovarian cancer patients. The aim of this study was to verify whether the expression of the analysed LUM gene and protein that was observed in tissue culture cell lines could also be confirmed in real cancer patient tissues. We analysed distinct types of ovarian cancer in which we could observe different expression levels of the LUM protein. A strong reaction was observed in stroma but not in cancer cells of serous adenocarcinoma patients (Figure 7A). In ovarian endometrioid adenocarcinoma patients, we could observe moderate to strong positive reactions in both the stroma and in the cytoplasm of cancer cells (Figure 7B).

## DISCUSSION

The most important problem with chemotherapy is the resistance of cancers to cytotoxic agents. Although some cancers are resistant to chemotherapy at the beginning of treatment, most develop resistance during treatment. For many years, researchers focused on cellular mechanisms of drug resistance [35]. Although these mechanisms are important, especially for *in vitro* studies, their significance in the clinic can be limited. In tumour tissue, other mechanisms are present that protect tumour cells against chemotherapy [9, 10]. These mechanisms are related to the architecture of the tumour and the tumour microenvironment [6, 8]. In tumour tissues, the expression of many ECM molecules, including small proteoglycan like LUM, was observed [36]. The expression of ECM molecules was not limited to tumour stroma and cancer-associated fibroblasts (CAF) but was also observed in cancer cells [9, 10, 15] and seems to play an important role in CAM-DR both *in vivo* [12] and *in vitro* [11]. The expression of some ECM components, such as collagen, was related to the resistance of tumours to chemotherapy [37]. This raises the question of whether ECM molecules can also be expressed in cancer cells in *in vitro* conditions and, even more importantly, whether they can play a role in resistance to chemotherapy. Thus, along with others, we used an RNA microarray to detect the expression of many ECM molecules in ovarian and breast cancer cell lines [13-16], and one of these molecules was LUM. Because LUM expression seems to be related to the progression of different cancers [21, 24-27], we decided to investigate its expression in drug-resistant ovarian cancer cell lines in more detail.

We observed increased expression of *LUM* in the cell lines resistant to DOX (W1DR), TOP (W1TR) and VIN (W1VR); however, although all these cell lines expressed much higher *LUM* transcript levels than the control, the differences between the W1DR and W1VR cell lines and the W1TR cell line was enormous. An increase greater than 1200-fold in the *LUM* transcript level suggests that overexpression of this gene can play a very important role in resistance to TOP. Immunofluorescence analysis confirmed increased expression of LUM in drug-resistant cell lines, which indicates that LUM is equally expressed in all cells. Because LUM can be present in the cell as a core protein [21], a glycoprotein [20], or a proteoglycan [22] and it can be secreted from the cell, we decided to investigate which molecular forms of LUM are present in cells and which forms are secreted into the cell culture media. In cell lysates, the most abundant form of LUM seems to be the form with a molecular mass of approximately 100 kDa. This form was overexpressed in VIN resistant cell line. In DOX resistant cell line, the LUM forms with molecular masses of 150 kDa and greater than 280 kDa seem to be overexpressed. In contrast, the TOP resistant cell line was characterized by an overexpression of the 50 kDa form of LUM. This result suggests that the expression of different LUM forms could be specific to resistance to different cytotoxic drugs. However, expression of LUM at the protein level in cell lysates did not fully reflect increased expression at the transcript level, especially in the W1TR cell line. Thus, we decided to check whether LUM could be secreted into the cell culture media. Indeed, in the cell culture medium, we observed enormous amount of the 50 kDa form of LUM in the W1TR cell line. However, we should emphasize that the exposure of the Western blot from the medium was much shorter compared to the exposure from the cell lysate. Similar to our study, Matsuda et al. [21] and Yamamoto et al. [38] also observed LUM proteins with different molecular masses in lung and pancreatic cancer cell lysates and corresponding culture media. Like in our study, the most abundant form of LUM in cell culture medium from the A549 lung cancer cell line had a molecular mass of 50 kDa [21]. This form was also the most abundant form of LUM in cell lysates from the MIA PaCa-2 pancreatic cancer cell line [26]. The increased molecular mass of LUM can result from diverse types of glycosylation. To clarify whether N-glycosylation, reported in many cancers and cancer cell lines [21, 38], could also be responsible for the increased mass of LUM, we performed digestion with PGNase F. Between different LUM bands, we observed the disappearance of those with molecular masses of 100 kDa and 50 kDa. Because the 100 kDa band was weakly detected in digestion experiments, we did not observe any additional bands after digestion. In contrast, the disappearance of the 50 kDa band and the appearance of the 38 kDa band, both from cell lysates and culture medium, was observed. This indicates that the 50 kDa form of LUM is the N-glycosylated form. A similar reduction of the molecular mass from 50 kDa to 38 kDa after digestion was also observed in the A549 lung cancer cell line [21]. PGNase F treatment did not change the mass of bands corresponding to 55, 150 and 280 kDa, suggesting that these masses could have resulted from O-glycosylation or KS attachment [20]. In summary, the most abundant expression of LUM was observed in the W1TR cell line that is resistant to TOP, and the most abundant form of LUM was the secreted N-glycosylated form, which had a mass of 50 kDa.

Very high expression of the secreted form of LUM could have resulted from very high level of COL3A1 expression in the W1TR cell line [15]. Previously, we reported the expression of several collagens in drug-resistant ovarian cancer cell lines. Among them, COL3A1 was the most abundantly expressed in the W1TR cell line. COL3A1 was not only present in cells but was also secreted from the cells and formed a structure similar to a spider’s web [15]. Here, we showed co-expression of COL3A1 and LUM in W1TR cells and confirmed the presence of secreted COL3A1 in the corresponding medium of W1TR cells. COL3A1 is a type III collagen and belongs to the fibrillar class [39]. It has been reported that LUM co-localizes with fibrillar collagen and plays an important role in the assembly and diameter of collagen fibres [23]. Collagen triple helix formation occurs in the endoplasmic reticulum. Next, pro-collagen molecules are secreted from the cell, and collagen fibril formation involving LUM occurs in the extracellular matrix [23, 39]. LUM deficiency leads to altered collagen morphology and to the presence of thicker fibrils [23]. Thus, very high expression of LUM in the W1TR cell line could be a result of its role in COL3A1 assembly.

The other possibility is that both COL3A1 and LUM play a role in TOP resistance in this cell line. The role of COL3A1 in drug resistance can result from direct binding of the drug molecule thus limiting drug diffusion and the activation of CAM-DR [15]. It is possible that secreted LUM plays a similar role in TOP resistance. High LUM protein levels in cell culture media can bind TOP molecules and inhibit their penetration into cancer cells. It was observed that the interaction of cancer cells with their microenvironment through surface receptors, such as integrins, can lead to the inhibition of drug-induced apoptosis. The interaction of pancreatic cancer cell lines with ECM molecules leads to CIS, 5-fluorouracil (5-FU) and DOX resistance [40]. The direct interaction of LUM with the α2β1 integrin expressed in melanoma has been reported [41, 42]. Thus, it seems possible that secreted LUM can interact with cell surface receptors and inhibit drug-induced apoptosis. Looking through literature data, we only found one paper concerning LUM expression in drug-resistant cell lines. Yamano et al. observed an increased expression of LUM in -CIS- resistant HNSCC cell lines [27]. Furthermore, silencing of *LUM* expression using siRNA led to a higher sensitivity to CIS, suggesting LUM plays a role in CIS resistance. In HNSCC tissue samples, the expression of LUM was significantly higher in patients that were not responding to CIS-based combination chemotherapy, confirming the role of LUM in drug resistance [27]. However, the authors of this study do not try to explain the mechanism of this resistance. In contrast to our study, they did not investigate the presence of secreted LUM. Our results suggest that the secreted form of LUM plays a dominant role in drug resistance.

Most papers concerning drug resistance are based on the comparison of drug-sensitive and drug-resistant pairs of cell lines. Researchers are mainly interested in the “established” mechanism of drug resistance. It is difficult to find papers concerning cancer cell responses to cytotoxic drugs during the first days of drug exposure. Recently, we published a paper describing the expression of ABC drug transporters and other genes during early response to TOP treatment [43]. Here, we observed a dose- and time-dependent increase of *LUM* mRNA in response to TOP. Increased expression in the first days after contact with cytotoxic drugs confirms that LUM can indeed be involved in TOP resistance.

Research performed on a small group of ovarian cancer patients showed different patterns of LUM protein expression according to the type of cancer. The expression of LUM was only observed in the stroma of some types of cancer, whereas in others, we observed moderate to strong reactions in both the stroma and cancer cells. Similar observations were made by other researchers. In lung ADC and SCC, the expression of LUM was observed both in cancer cells and in stroma, although their levels of expression and their correlation to clinical data were different. In SCC, LUM expression was found in stromal tissue but not in the cytoplasm of cancer cells, which was correlated with vascular invasion. In contrast, LUM expression in cancer cells correlated with pleural infusion and larger tumour sizes in ADC [21]. In advanced colorectal cancer, LUM expression was observed only in cancer cells and was correlated with metastasis in lymph nodes, the depth of tumour invasion and significantly lower survival rates [25]. In pancreatic cancer, the expression of LUM was observed both in cancer cells as well as in stroma. However, only the stromal expression was correlated with clinicopathological factors such as advanced stages of cancer and shorter lengths of survival time of patients [26].

## MATERIALS AND METHODS

### Reagents and antibodies

DOX, TOP, and VIN were obtained from Sigma (St. Louis, MO). RPMI-1640 medium, foetal bovine serum, antibiotic-antimycotic solution, and L-glutamine were also purchased from Sigma. Rabbit polyclonal anti-lumican Ab was obtained from Abnova (Taipei, Taiwan). Goat anti-rabbit horseradish peroxidase- (HRP) conjugated Ab was purchased from Santa Cruz Biotechnology (Santa Cruz, CA, US). The MFP488 fluorescent secondary antibody was obtained from MoBiTec (Goettingen, Germany). The Cy3-conjugated fluorescent secondary antibody was obtained from, Jackson ImmunoResearch (West Grove, PA, USA). The mounting medium with DAPI was obtained from Santa Cruz Biotechnology (Santa Cruz, CA, US). Columns for protein isolation from serum were purchased from Merck Millipore (Billerica, MA, US).

### Cell lines and cell culture

The human primary ovarian cancer cell line W1 was established using ovarian cancer tissue obtained from an untreated patient. W1 sublines resistant to DOX [W1DR (W1 doxorubicin-resistant)], TOP [W1TR (W1 topotecan-resistant)], and VIN [W1VR (W1 vincristine-resistant)] were obtained by exposing W1 cells to the drugs at incrementally increasing concentrations. All resistant cell lines were generated in our laboratory. The cells were seed at 10000 cells/cm^2^ in 25 cm^2^ flasks in RPMI-1640 medium supplemented with the appropriate drug. The established concentrations of the initial drug exposures were 10 ng/mL for DOX, 0.5 ng/mL for TOP and 0.5 ng/mL for VIN. Each cell line was exposed three times for 3-day periods during a 3-6 week period and growth recovery between cycles was allowed. The drug concentrations were doubled after the completion of three cycles and the procedure was repeated until the final drug levels were achieved. The final concentrations used for selecting the resistant cells were 100 ng/ml for DOX, 24 ng/ml for TOP and 10 ng/ml for VIN. These concentrations were two-fold higher than their respective plasma concentrations 2 hours after intravenous administration. The drug sensitivities of the sensitive and drug-resistant cell lines were confirmed by the MTT cell survival assay. The increase in resistance according to parental drug sensitive cell lines were as follow: 10.3 fold for W1DR vs W1 (IC50 – 215 ng/ml and 20.8 ng/ml, respectively); 20 fold for W1TR vs W1 (IC50 – 83.9 ng/ml and 4.19 ng/ml, respectively) and 24.5 fold for W1VR vs W1 (IC50 – 45.3 ng/ml and 1.85 ng/ml, respectively) as described previously [2].

### Examination of gene expression using QPCR

The changes in *LUM* expression in the W1 and drug-resistant cell lines were examined. RNA was isolated using the GeneMATRIX Universal RNA purification kit (EURx Ltd. Gdansk, Poland) as described by the manufacturer’s protocol. Reverse transcription was performed using M-MLV reverse transcriptase (Invitrogen) and a thermal cycler (Veriti 96-well Thermal Cycler) as described in the manufacturer’s protocol. Two micrograms of RNA was used for cDNA synthesis. Real-time PCR was performed using the 7900HT Fast Real-Time PCR System (Applied Biosystems), Maxima SYBR Green/ROX qPCR Master Mix (Fermentas) and the sequence-specific primers that are indicated in Table 1. Glyceraldehyde-3-phosphate dehydrogenase (*GADPH*), *β-actin*, hypoxanthine-guanine phosphoribosyltransferase 1 (*HRPT1*) and beta-2-microglobulin (*β2M*) served as the normalizing genes (geometric mean) for the gene expressions being analysed. Gene expressions were analysed using the relative quantification (RQ) method. The RQ method estimates the differences in gene expression against a calibrator (drug-sensitive line) (RQ of the calibrator = 1). The drug-sensitive W1 cell line was used as the calibrator. The analysis was conducted using the following standard formula: RQ = 2 − ΔΔCt (where ΔΔCt = ΔCt of the sample (drug-resistant line) − ΔCt of the calibrator (drug sensitive line)). The graphs were plotted using Sigma Plot. For amplification, 12.5 μL of Maxima SYBR Green/ROX qPCR Master Mix (Fermentas), 1 μL of each primer (Oligo, Warsaw, Poland) (Table 1), 9.5 μL of water, and 1 μL of cDNA solution were mixed together. One RNA sample from each preparation was processed without the RT-reaction to provide a negative control in the subsequent PCR reaction. Sample amplification included a hot start (95°C, 15 min) followed by 40 cycles of denaturation at 95°C for 15 seconds, annealing at 60°C for 30 seconds, and extension at 72°C for 30 seconds. After amplification, melt curve analysis was conducted to analyse the product melting temperatures. The amplification products were also resolved using 3% agarose gel electrophoresis and visualized by ethidium bromide staining.

**Table 1:** Oligonucleotide sequences used for RQ-PCR analysis

Transcript	Sequence (5’-3’ direction)	ENST number http://www.ensembl.org	Product size (bp)
LUM	CCTGGTTGAGCTGGATCTGT CCCCAGGATCTTGCAGAAG	00000266718	133 bp
GADPH	GAAGGTGAAGGTCGGAGTCA GACAAGCTTCCCGTTCTCAG	00000229239	199 bp
β-actin	TCTGGCACCACACCTTCTAC GATAGCACAGCCTGGATAGC	00000331789	169 bp
HRPT1	CTGAGGATTTGGAAAGGGTG AATCCAGCAGGTCAGCAAAG	00000298556	156 bp
β2M	CGCTACTCTCTCTTTCTGGC ATGTCGGATGGATGAAACCC	00000558401	133 bp

### Protein isolation from cell culture and media and glycosidase treatment

The cells (1 × 10^6^ cells/25 μL lysis buffer) were lysed in RIPA buffer containing protease inhibitor cocktail (ROCHE) for 60 min on ice at 4°C. The lysates were centrifuged at 12000 × g for 15 min at 4°C, and protein concentrations were determined using the BioRad (Hercules, CA) Bradford protein assay system. To isolate proteins from media, cells were cultured in serum-free media for 72 hours. Next, the media was centrifuged at 15 000 rpm for 30 min at RT. Then, the supernatants were transferred to Amicon Ultra-15 3K centrifuge filter devices and centrifuged according to the manufacturer’s instructions (60 min, 4 000 x g, RT, swinging-bucket rotor). Glycosidase treatment was performed by incubating 35 μg of cell lysate with 5 μl of PNGase F, 0.5 μl of β-ME and 1 μl of SDS at 37°C for 10 min.

### SDS-PAGE and western blot analysis of LUM

Thirty micrograms of protein from each sample was resuspended in 4 x loading buffer (BioRad) and incubated at RT for 20 min. The resuspended protein was loaded into each well and separated on a 4-20% mini-PROTEAN^®^ TGX™ precast gel using the SDS-PAGE technique. The proteins were transferred to a nitrocellulose membrane, blocked with 5% milk in TBS/Tween (0.1 M Tris-HCl, 0.15 M NaCl, 0.1% Tween 20) and immunodetected using rabbit anti-LUM Ab at 1:1000 dilution and the appropriate HRP-conjugated secondary Ab. Chemiluminescence detection of the separated bands was performed using an enhanced chemical luminescence (ECL) kit (Femto Super Signal Reagent) and Hyperfilm ECL from Amersham (Piscataway, NJ). To normalize protein loading in the lanes, the membranes were stripped and reblotted with rabbit anti-GADPH Ab, from Santa Cruz Biotechnology, at a 1:1000 dilution and goat anti-rabbit HRP-conjugated Ab.

### Immunofluorescence analysis

The cells were cultured on microscopic glass slides and grown to a near-confluent state. Afterwards, the cells were fixed in 4% PFA in PBS for 10 min at room temperature, permeabilized in ice-cold acetone/methanol (1:1) for 10 min at -20°C, rinsed with PBS and blocked in 3% BSA for 45 min. Anti-LUM primary antibody (1:200, rabbit monoclonal anti-LUM antibody, Abnova, Taipei, Taiwan) was used for detection along with the corresponding green dye-labelled secondary antibody (MFP488, donkey anti-goat IgG, 1:200, MoBiTec, Goettingen, Germany). Afterwards, the cells were washed three times with PBS and sealed with DAPI-containing mounting medium. The cells were viewed under a fluorescence microscope (Zeiss Axio-Imager.Z1).

The expression of LUM was analysed under a fluorescence microscope (Zeiss Axio-Imager.Z1) by pseudo-colour representations of fluorescence intensity for DAPI at 365 nm excitation and 420 nm emission wavelengths (blue) and for MFP488 at 470 nm excitation and 525 nm emission wavelengths (green).

### Double immunofluorescence analysis

For the double fluorescence staining, the fixation, blocking and washing steps were conducted as described above. Incubation with the mixture of two primary antibodies for LUM [(1:200, rabbit monoclonal anti-LUM antibody, Abnova, Taipei, Taiwan) and COL3A1 (1:100, goat polyclonal anti-COL3A1 antibody, Santa Cruz Biotechnology, Dallas, TX, USA)] proceeded at 4°C overnight. The cells were then washed with PBS and incubated with the mixture of the two respective green dye-labelled (MFP488, donkey anti-goat IgG, 1:200, MoBiTec, Goettingen, Germany) and red dye-labelled (Cy3, donkey anti-rabbit IgG, 1:200, Jackson ImmunoResearch, West Grove, PA, USA) secondary antibodies for 1 hour at room temperature. Afterwards, the cells were washed three times with PBS and sealed with DAPI-containing mounting medium. The expression of LUM and COL3A1 was analysed under a fluorescence microscope (Zeiss Axio-Imager.Z1) by pseudo-colour representations of fluorescence intensity for DAPI at 365 nm excitation and 420 nm emission wavelengths (blue), for Cy3 at 550 nm excitation and 605 nm emission wavelengths (red) and for MFP488 at 470 nm excitation and 525 nm emission wavelengths (green).

### Incubation of cells with TOP

In time-course experiments, the W1 line was treated with TOP at a concentration of 10 ng/ml and 20 ng/ml. The starting cell concentration was 0.5 x 10^6^ in 1 ml of medium per well in 6 –well plates. The cell count and viability were determined before the cells were used in the different assays. Viability was determined by trypan blue exclusion criteria. At 24, 48 and 72 hours after treatment, the cells were harvested and used for RNA isolation.

### Immunohistochemistry

Immunohistochemical analysis was performed on transverse 5 μm formalin-fixed, paraffin embedded sections from human ovarian carcinomas placed on SuperFrost/Plus microscope slides. We investigated tissues from ovarian cancer patients. The analysis of LUM expression was performed using the polymer-based immunohistochemical (IHC) technique [34] and the specific primary antibody (rabbit polyclonal anti-LUM antibody, 1:200, Abnova, Taipei, Taiwan).

The slides were dewaxed with xylene and gradually hydrated. The activity of endogenous peroxidase was blocked by a 30 min exposure to 1% H_2_O_2_. The sections were incubated with the primary antibodies overnight at 4°C followed by incubation with EnVision Detection System Peroxidase/DAB, Rabbit/Mouse for 30 min (Dako REALTMEnVisionTM Detection System peroxidase/DAB+, Rabbit/Mouse, Dako, Glostrup, Denmark). The sections were then counterstained with haematoxylin, dehydrated and mounted.

Histological slides with expressed proteins were examined under an optical Olympus BH-2 microscope coupled to a digital camera. Colour microscope images were recorded using LUCIA Image 5.0 computer software (Nikon, Tokyo, Japan).

The expression of the analysed marker (only clearly labelled cells with a cytoplasmic signal were considered) was calculated by considering the mean proportion of immunopositive cancer cells among all cancer cells that were counted in 10 light microscope fields that were each at magnification of 400x (at least 100 cancer cells per microscopic field). Expression was evaluated using the semi-quantitative scale in which a score of 0 (negative) corresponded to no observed staining or less than 10% of cancer cells with weak positivity, a score of 1 (weak) corresponded to 11% to 50% positive cancer cells, a score of 2 (moderate) corresponded to 51% to 75% positive cancer cells, and a score of 3 (strong) corresponded to up to 75% positive cancer cells.

### Statistical analysis

Statistical analysis was performed using Microsoft Excel software. The statistical significance of the differences was determined using the Student’s t-test, and p-values of 0.05 or less were considered statistically significant.

## CONCLUSIONS

In summary, our results present the expression of LUM at the mRNA and protein levels in drug-resistant ovarian cancer cell lines and their corresponding media. Our results suggest that the LUM protein can be implicated in drug resistance. Co-expression of LUM with COL3A1 also suggests that LUM plays an important role in COL3A1 assembly in the TOP-resistant cell line. The presence of extracellular LUM and COL3A1 suggest that CAM-DR can also play a role in drug resistance in cells that grow as a monolayer. Detection of LUM in ovarian cancer tissue confirms its role in cancer pathogenesis. The significance of LUM expression in cancer cell drug resistance and cancer development requires further investigation and should be confirmed in other ovarian cancer cell lines, a large cohort of clinical specimens and in animal studies.
